# Effects of Fortified Formula Milk Supplementation on Neurocognitive Development and the Microbiota–Gut–Brain Axis in Preschool Children: A Cluster-Randomized, Double-Blind, Controlled Trial

**DOI:** 10.3390/nu18071167

**Published:** 2026-04-07

**Authors:** Yifan Gong, Xingwen Zhao, Qi Zhang, Xinxin Yan, Bin Sun, Xinyi Li, Qixu Han, Yiran Guan, Huiyu Chen, Meina Li, Jie Guo, Biao Liu, Ran Wang, Baotang Zhao, Yan Zhang, Jingjing He

**Affiliations:** 1 College of Food Science and Engineering, Gansu Agricultural University, Lanzhou 730070, China; 1073323120744@st.gsau.edu.cn (Y.G.); zhaobaotang@gsau.edu.cn (B.Z.); 2 Key Laboratory of Precision Nutrition and Food Quality, Department of Nutrition and Health, China Agricultural University, Beijing 100193, China; zhangqi@cau.edu.cn (Q.Z.); yxx2025331001@cau.edu.cn (X.Y.); sy20243313908@cau.edu.cn (X.L.); hqx@cau.edu.cn (Q.H.); b20253311579@cau.edu.cn (Y.G.); b20243311375@cau.edu.cn (H.C.); 15122295019@163.com (M.L.); guojie@cau.edu.cn (J.G.); wangran@cau.edu.cn (R.W.); 3 Dali Vocational and Technical College of Agriculture and Forestry, Dali 671003, China; 13608823221@139.com; 4 National Technology Innovation Center for Dairy, Hohhot 010110, China; sunbin11@yili.com; 5 Milk Powder BU, Inner Mongolia Yili Industrial Group Co., Ltd., Hohhot 010110, China; bliu@yili.com

**Keywords:** fortified formula milk, preschool children, cognitive function, processing speed, gut microbiota, metabolomics, microbiota–gut–brain axis

## Abstract

Background/Objectives: The preschool period is critical for neurodevelopment, yet evidence investigating fortified formula’s effect and potential microbiota–gut–brain axis mechanisms in this age group is limited. To evaluate fortified formula milk’s effect on neurodevelopment and explore potential microbiota–gut–brain axis mechanisms in preschool children. Methods: In this 9-month cluster-randomized, double-blind, controlled trial, 120 healthy children aged 3–6 years from four kindergarten classes were stratified by grade and randomly allocated (1:1) to receive either multi-nutrient fortified formula (intervention, *n* = 60) or standard control milk (*n* = 60). Neurocognitive function was assessed using the Wechsler Preschool and Primary Scale of Intelligence, Fourth Edition (WPPSI-IV). Safety was evaluated through anthropometry and blood biochemistry. Gut microbiota (16S rRNA sequencing) and fecal metabolomes (untargeted LC-MS) were analyzed at baseline and 9 months. Results: The intention-to-treat (ITT) analysis showed no significant difference in Full Scale Intelligence Quotient (adjusted mean difference: 1.05 points; 95% CI: −1.42, 3.52; *p* = 0.400). However, the intervention group significantly improved the Processing Speed Index (adjusted mean difference: 5.91 points; 95% CI: 1.88, 9.93; *p* = 0.004), increased gut microbial alpha diversity (Shannon index) and *Bifidobacterium* abundance. Metabolomic analysis revealed elevated fecal 2-hydroxybutyric acid (2-HB), a marker of propanoate metabolism. Increases in both *Bifidobacterium* and 2-HB levels showed a positive association with PSI improvement (both *p* < 0.05). All children maintained normal growth and safety parameters. Conclusions: Fortified formula milk improved processing speed in preschoolers, a benefit associated with gut ecosystem modulation characterized by *Bifidobacterium* enrichment and upregulated microbial propanoate metabolism. These results offer preliminary evidence for the role of the microbiota–gut–brain axis in nutritional cognitive programming during early childhood. (Clinical Trial Registry: ChiCTR2400084211).

## 1. Introduction

The preschool phase, spanning ages 3 to 6 years old, constitutes a pivotal window of rapid neurocognitive development and a vital transition from home to school, preparing children for formal education. During this stage, core executive functions such as working memory and attentional control develop rapidly, laying the foundation for future academic and social adaptation [1,2]. Neuroimaging and empirical studies indicate that cortical expansion and white matter myelination occur highly synchronously during these years [3]. These structural changes provide the neural basis for efficient early information processing and are closely linked to improvements in children’s behavioral performance and cognitive abilities [4]. Consequently, the 3–6 age range is recognized as a pivotal window for assessing the impact of external factors on neurocognitive development.

Brain development is highly sensitive to nutritional requirements. Beyond macronutrients, micronutrients such as iron, zinc, choline, docosahexaenoic acid (DHA), arachidonic acid (ARA), and B vitamins play crucial roles in neuronal proliferation, differentiation, and neurotransmitter synthesis [5,6]. However, preschoolers’ dietary habits remain unstable, with picky eating and selective eating being common, potentially leading to micronutrient deficiencies and creating a phenomenon of “hidden hunger.” Global surveys indicate that approximately 56% (about 372 million) of preschool children faced micronutrient deficiency risks between 2003 and 2019 [7]. These findings suggest potential nutritional risks affecting neurocognitive development during the preschool years.

Systematic reviews [8] and several clinical trials [9,10] have demonstrated that nutritional interventions may enhance children’s cognitive function. Formula milk, designed as a comprehensive food matrix to deliver multiple key nutrients, offers a practical solution for addressing nutritional gaps [11] and has shown potential for improving cognitive outcomes [9,12]. In recent years, some formula products have further incorporated functional ingredients such as probiotics and prebiotics. This development has led to an extended hypothesis: the potential benefits of formula milk for neurodevelopment may extend beyond direct nutrient provision to include modulation of the “microbiota-gut-brain axis.” Research on animals and some infant intervention research indicate that gut microbiota can communicate bidirectionally with the central nervous system through immune and endocrine signaling, as well as via neuro-regulatory pathways involving the vagus nerve, thereby influencing brain function and behavioral regulation [13,14,15].

Despite this promising mechanistic hypothesis, critical evidence gaps remain regarding its applicability to healthy preschool-aged children. First, high-quality randomized controlled trials (RCTs) involving long-term interventions with compound formula milk in this specific age group are scarce, with most evidence derived from studies in infants [16] or focused on single nutrients [17]. Second, existing studies predominantly focus on overall cognitive scores or physical indicators, with limited detailed assessments of their specific effects on cognitive subdomains [18]. Furthermore, the “microbiota-gut-brain axis” as a potential pathway for formula milk’s effects has not been prospectively examined in preschool children using multidimensional biological evidence.

To address these gaps, we conducted a 9-month, cluster-RCT. The primary objective was to evaluate whether long-term consumption of a multi-nutrient fortified formula (containing probiotics) improves global and domain-specific neurocognitive performance in 3- to 6-year-old children. Simultaneously, we sought to explore the possible role of the microbiota–gut–brain axis by profiling intervention-induced shifts in the intestinal microbiome and fecal metabolome, and by linking them to cognitive performance.

## 2. Materials and Methods

### 2.1. Study Design

The present research utilized a cluster-randomized, double-blind, parallel-group, controlled design. The cluster design (randomization at the class level) was adopted to minimize the risk of intervention contamination (e.g., sharing of milk between children) and to facilitate practical administration within the kindergarten setting. Ethical clearance for the experimental protocol and consent documentation was granted by the Human Research Ethics Committee of China Agricultural University before data collection began (Approval No.: CAUHR-20240102; approved on 2 January 2024). The clinical trial registration number of this research was ChiCTR2400084211 (registered on 13 May 2024; URL: https://www.chictr.org.cn/showproj.html?proj=228045, accessed on 6 April 2026). Fieldwork took place in Yunnan Province, China, in full compliance with the Declaration of Helsinki [19] and applicable national standards governing clinical research.

**Figure 1:**
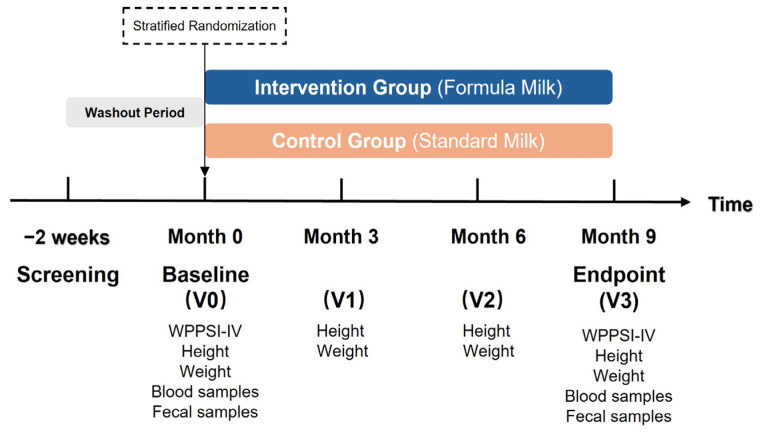
Study design. Participants were stratified-randomized to receive formula milk or standard milk. Anthropometric measurements were monitored every 3 months. WPPSI-IV testing and biological sample collection were performed at baseline and endpoint. Abbreviations: WPPSI-IV, Wechsler Preschool and Primary Scale of Intelligence-Fourth Edition.

Figure 1 outlines the experimental design. Participants first underwent a two-week washout period, during which they were required to abstain from probiotics and fermented foods (e.g., kimchi, yogurt, and live-culture lactic acid beverages). Following the washout period, baseline assessments were performed, comprising the Wechsler Preschool and Primary Scale of Intelligence, Fourth Edition (WPPSI-IV), anthropometric measurements (height and weight), and the collection of fecal and blood samples. Subsequently, the 9-month intervention phase began, with the intervention group consuming a fortified formula milk and the control group consuming standard milk. Throughout this period, participants maintained their habitual dietary, exercise, and sleep routines while continuing to abstain from fermented foods. The research team conducted follow-up visits every 3 months to monitor growth indicators. At the conclusion of the intervention (Month 9), a final assessment identical to the baseline was performed, including the WPPSI-IV test, anthropometric measurements, and biological sample collection. The reporting of this study conforms to the CONSORT 2025 statement (see Supplementary Materials).

### 2.2. Study Participants

This study commenced in July 2024 at a kindergarten in Binchuan County, Yunnan Province, China. We enrolled 120 preschool children between the ages of 3 and 6 were recruited from four classes, including three senior classes and one middle class. All participants fulfilled the specified eligibility criteria.

Criteria for Inclusion: (1) Healthy preschool children between the ages of 3 and 6; (2) Willingness to consume the study product during the research period; (3) Signed informed consent obtained from parents or legal guardians. Criteria for Exclusion: (1) Children with severe malnutrition, food allergies, other allergic diseases, or lactose intolerance; (2) Children with gastrointestinal diseases, or disorders of the cardiovascular, hepatic, renal, or hematopoietic systems, neuropsychiatric conditions, or a history of surgery; (3) Parents unwilling to adhere to the study protocol; (4) Mothers with a history of psychiatric illness or use of anti-anxiety or antidepressants during pregnancy; (5) Children currently participating in another clinical trial.

### 2.3. Randomization, Allocation Concealment, and Blinding

An independent statistician, who had no involvement in participant recruitment or subsequent trial conduct, performed the randomization using a computer-generated random sequence. Given the strong correlation between age and cognitive development, stratification was applied at the grade level. A mixed randomization approach was adopted for practical management: two senior classes were randomized as intact clusters (one to each group). For the remaining senior class and the middle class, individual randomization was performed within each class. This resulted in 60 children per group, with identical distributions of senior and middle kindergarten children (45:15).

To ensure allocation concealment, the randomization sequence (indicating group assignment for each class and, where applicable, individual child) was kept in sealed, opaque envelopes by the kindergarten principal, who was not involved in outcome assessment. The envelopes were opened only after all baseline data were collected and locked, and just before the commencement of the intervention delivery. This process ensured that researchers conducting baseline assessments and participants were unaware of future group assignments.

Throughout the study, participants, guardians, outcome evaluators, and statisticians were masked to the group allocations.

### 2.4. Research Products

Following baseline assessments, the 9-month intervention was initiated. The intervention group received formula milk, while the control group received standard milk. In contrast to the control product, the intervention formula was fortified with DHA (50 mg/100 g), ARA (60 mg/100 g), and multiple vitamins and minerals. Notably, it was enriched with a specific probiotic blend capable of modulating gut microbiota, comprising *Bifidobacterium animalis* subsp. *lactis* HN019 (concentration ≥ 4 × 10^6^ CFU/g) and *Bifidobacterium animalis* subsp. *lactis* BL-99 (concentration ≥ 2 × 10^6^ CFU/g), alongside prebiotic complexes including galacto-oligosaccharides (GOS) and isomerized lactose. Furthermore, the formula was further fortified with lactoferrin and yeast β-glucan to synergistically support neurostructural development and immune function (see Table 1 for a detailed comparison of key nutritional components).

Both products were administered twice daily at 10:00 and 15:00. Milk preparation was centralized and managed by unblinded staff members who were independent of the outcome assessment and data analysis processes. Standardized servings were formulated by mixing 25 g of milk powder with 160 mL of warm water for each group. Accordingly, each child in the intervention group received 50 g of formula milk powder per day, corresponding to a daily intake of *B. lactis* HN019 ≥ 2 × 10^8^ CFU/day and *B. lactis* BL-99 ≥ 1 × 10^8^ CFU/day. The reconstituted milk was distributed in uniform cups labeled with the child’s class and identification number. Class teachers distributed the milk according to these IDs and supervised the entire consumption process to prevent cross-consumption between groups. Additionally, to minimize confounding factors from external microbial sources, all participants were instructed to abstain from other fermented foods (e.g., yogurt) or probiotic products throughout the intervention period. Compliance with this instruction was monitored through monthly parent-reported questionnaires and telephone follow-ups. In this study, adherence was defined as consuming no more than one serving per month of any restricted food.

### 2.5. Study Outcomes

#### 2.5.1. Primary Outcome

The primary outcome was the Full-Scale Intelligence Quotient (FSIQ) derived from the Chinese version of WPPSI-IV. The WPPSI-IV is a widely used standardized cognitive assessment tool for preschool children with sound psychometric properties. Data from the Chinese version’s revised manual [20] indicate that the scale demonstrates robust construct validity across the full scale and all dimensional indices. Furthermore, its core metrics—including test–retest reliability and validity—meet the fundamental requirements for clinical cognitive assessment.

To ensure the reliability of assessment results, all five examiners underwent systematic training on the WPPSI-IV and passed practical assessments prior to formal testing. Cognitive evaluations were conducted in separate, well-lit rooms free from distractions, with participation limited to the examiner and the child during the assessment. The administration process strictly adhered to standardized procedures: the examiner sat across from the child at a table, observing and fully documenting behavioral responses in real time. Assessments were conducted in single sessions whenever possible. In cases of interruption due to special circumstances, the examiner strictly followed the Start and Reverse rules outlined in the test manual to ensure data validity upon restarting the assessment.

The WPPSI-IV consists of 13 subtests, from which FSIQ was derived as the primary outcome measure. In interpreting the results, higher scores indicate better overall cognitive functioning [21].

#### 2.5.2. Secondary Outcomes

##### Cognitive Subdomains

In addition to the primary outcome, the WPPSI-IV provides five core indices that were prespecified as secondary outcomes: Verbal Comprehension Index (VCI), Visual–Spatial Index (VSI), Fluid Reasoning Index (FRI), Working Memory Index (WMI), and Processing Speed Index (PSI). The assessment procedures for these indices followed the same standardized protocol described above for the primary outcome.

##### Growth Outcomes

Height and weight were measured at four time points: at baseline, as well as during the 3-, 6-, and 9-month post-intervention assessments. To minimize the impact of diurnal variation, all measurements were performed by trained professionals within the same 2 h window each day.

Height was measured using a mechanical stadiometer (calibrated with a steel ruler; error ≤ 0.2%). Participants stood barefoot without outer garments, maintaining a natural upright posture. Measurements followed standard anthropometric protocols and were documented with a precision of 0.1 cm. Body mass was assessed using a lever-type balance calibrated with standard weights (error ≤ 0.1%). Participants were weighed barefoot while wearing light clothing, with readings recorded to the nearest 0.1 kg.

##### Blood Parameters

Every participant was asked to voluntarily contribute blood specimens. Each sampling was conducted in a fasting state, with researchers drawing 5–8 milliliters of blood samples from the antecubital vein. This sampling procedure was performed twice during the study period (at months 0 and 9).

After processing the blood samples, the measurements of various parameters were strictly performed according to the instrument and reagent manuals. Complete blood counts were determined via the Sysmex XN-9000 fully automated hematology system (Sysmex Corp., Kobe, Japan). Liver and kidney function parameters were measured using enzymatic and kinetic assays, both performed on a fully automated biochemical analyzer (Cobas c 702, Roche Diagnostics, Tokyo, Japan).

##### Gut Microbiota and Metabolome Analysis of Fecal Samples

Before the trial, stool specimens were obtained from 30 individuals chosen at random from each study arm. These participants also provided post-intervention samples, resulting in an aggregate of 120 specimens. Early morning stool collection was performed under fasting states using sterile containers. The samples were promptly chilled on ice, delivered to the research facility within 60 min, and cryopreserved at −80 °C until analyzed [22].

Total genomic DNA from stools was isolated utilizing the FastPure Stool DNA Isolation Kit (MJYH, Shanghai, China), with concentrations subsequently quantified via a NanoDrop 2000 spectrophotometer (Thermo Fisher Scientific, Waltham, MA, USA). The 16S rRNA gene’s V3–V4 hypervariable region was targeted for amplification using primers 338F (5′-ACTCCTACGGGAGGCAGCAG-3′) and 806R (5′-GGACTACHVGGGTWTCTAAT-3′). Thermal cycling parameters were set as follows: 3 min of initial denaturation at 95 °C; 27 cycles consisting of 95 °C (30 s), 55 °C (30 s), and 72 °C (30 s); and a 10 min final extension at 72 °C. Shanghai Majorbio Bio-Pharm Technology (Shanghai, China) performed the library sequencing on an Illumina NextSeq 2000 platform (Illumina Inc., San Diego, CA, USA), along with preliminary raw data processing. Species-level annotation of amplicon sequence variants (ASVs) was achieved through the Naive Bayes classifier within the QIIME 2 framework [23]. Alpha diversity metrics (e.g., Chao and Shannon indices) were estimated employing the mothur software package (v.1.30.2) [24] (http://www.mothur.org/wiki/Calculators, accessed on 25 November 2025).

Fecal metabolite extraction and analysis followed published methods [25] with modifications tailored to the sample characteristics of this study. For sample detection, a Vanquish UHPLC platform (Thermo Fisher Scientific) was employed, featuring a Waters ACQUITY UPLC BEH Amide column (Waters Corporation, Milford, MA, USA) (2.1 mm × 50 mm, 1.7 μm) that was linked to an Orbitrap Exploris 120 mass spectrometer (Thermo Fisher Scientific) [26]. Mobile phase A was prepared by adding 25 mmol/L ammonium acetate and 25 mmol/L ammonium hydroxide into ultrapure water, whereas mobile phase B consisted of acetonitrile. Mass spectrometry data were acquired in IDA mode with a full scan resolution of 60,000, secondary resolution of 15,000, and collision energy set to SNCE 20/30/40 mode. Raw data underwent conversion via ProteoWizard (v.3.0.24054) [27], followed by peak extraction and alignment through the XCMS package (v.4.1.12) [28]. After total ion current (TIC) normalization, SIMCA (v.18.0.1) was utilized for OPLS-DA modeling, with 200 permutation tests ensuring model robustness. The BiotreeDB database was employed for metabolite identification, whereas pathway enrichment analysis relied on the KEGG (http://www.genome.jp/kegg/, accessed on 24 October 2025) and MetaboAnalyst (http://www.metaboanalyst.ca/, accessed on 24 October 2025) [29] platforms.

### 2.6. Adverse Events

Throughout the study period, monitoring of adverse events (AEs) was conducted by kindergarten classroom teachers. Teachers used a standardized Adverse Event Record Form to document health-related events occurring in participating children. Recorded information included key details such as clinical manifestations, time of onset and resolution, severity, treatments, and outcome. Additionally, records included antibiotic use during illness and the time of last use. The Principal Investigator (PI) periodically reviewed all records and assessed potential associations between adverse events and the study product based on predefined criteria. For adverse events persisting at study completion, the research team conducted follow-up until resolution or stabilization of the condition.

### 2.7. Sample Size Calculation and Statistical Analysis

The determination of sample size was driven by the pre-defined primary endpoint, specifically FSIQ from the WPPSI-IV, drawing from a comparable RCT [9] that showed an inter-group FSIQ variance of roughly 6.0 points (SD = 11.0). With the Type I error rate (α) set at 0.05 and a statistical power of 0.80, the analysis required at least 54 subjects in each arm. To compensate for a projected 10% dropout rate, 60 participants were enrolled per group, yielding an aggregate study population of 120 individuals. We acknowledge that this calculation did not adjust for the design effect inherent in the cluster-randomized design implemented in this study. Consequently, the present sample size is interpreted as adequate for an exploratory trial aimed at generating mechanistic hypotheses and estimating effect sizes to inform future definitive research.

All analyses adhered to the intention-to-treat principle. In descriptive analyses, continuous data following a normal distribution were expressed as mean ± SD, while non-normally distributed variables were reported as medians (25–75th percentiles). Frequencies and proportions [*n* (%)] were used to describe categorical data. Graphs were used to display means and their 95% confidence intervals (95% CI).

The primary analysis for neurocognitive outcomes (e.g., WPPSI-IV indices) employed a linear mixed-effects model using an analysis of covariance (ANCOVA) approach. The model specified the endpoint score (9-month) as the dependent variable, with treatment group as a fixed effect, the corresponding baseline score as a covariate, while incorporating class-level random intercepts to address the cluster randomization. This model directly yields the adjusted between-group mean difference at endpoint with its 95% confidence interval (CI). For the prespecified secondary outcomes, including the cognitive subdomains (VCI, VSI, FRI, WMI, PSI) as well as growth, safety, and multi-omics measures, all analyses were considered exploratory. For continuous secondary outcomes, between-group comparisons at follow-up time points were assessed via unpaired *t*-tests or Mann–Whitney U tests, depending on the data distribution. No adjustment for multiple comparisons was applied, and findings should be interpreted with caution.

For microbiome data analysis, β-diversity was visualized via PCoA based on Bray–Curtis distances, with PERMANOVA tests evaluating the explanatory power and statistical significance of grouping factors on community differences. Within-group shifts in genus-level relative abundance were evaluated with Wilcoxon signed-rank tests, whereas inter-group differences in these changes were analyzed using unpaired *t*-tests or Mann–Whitney U tests. Regarding metabolomics, TIC-normalized data underwent multivariate analysis via OPLS-DA modeling, with model stability confirmed through random permutation testing. We identified differential metabolites using a combined threshold of VIP > 1 and *p* < 0.05. Spearman’s rank correlation was applied to investigate the relationships among phenotypic markers, bacterial composition, and metabolic features. Statistical computations were carried out via IBM SPSS Statistics (v.28.0; IBM Corporation, Armonk, NY, USA), while data visualization was performed using GraphPad Prism 8 (GraphPad Software, San Diego, CA, USA). All statistical tests were two-sided, and significance was defined as *p* < 0.05.

## 3. Results

### 3.1. Study Population and Baseline Characteristics

**Figure 2:**
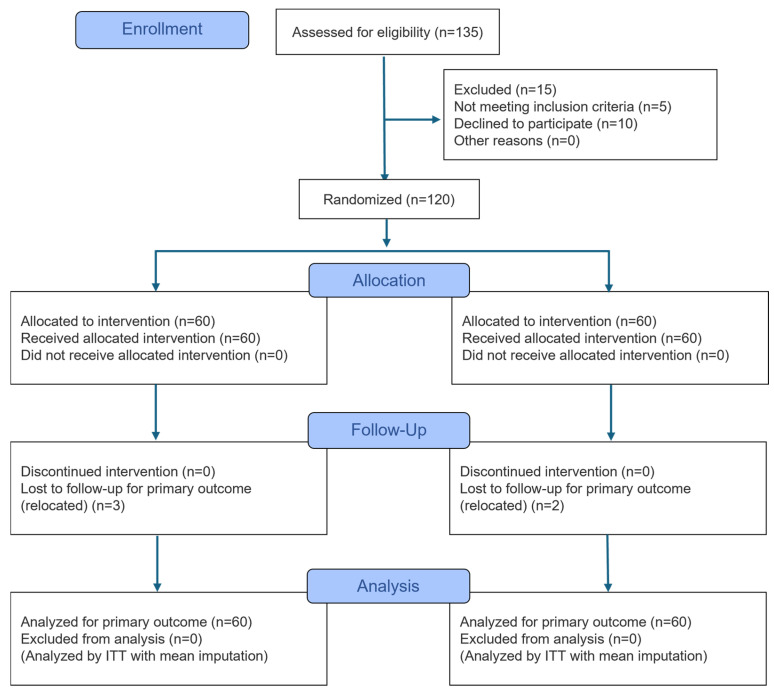
CONSORT 2025 flow diagram. Flow diagram illustrates the progress of participants through the phases of the 9-month cluster-randomized trial, including enrollment, allocation, follow-up, and data analysis using the intention-to-treat (ITT) principle.

In total, 120 children were recruited and assigned to either the intervention (*n* = 60) or the control arm (*n* = 60). Over the 9-month study period, five subjects were lost to follow-up because of family relocation or school changes (4.2% dropout rate). The intention-to-treat principle was applied to all randomized participants, with group mean imputation used for missing data. The CONSORT flow chart is illustrated in Figure 2.

Baseline characteristics for both study arms are summarized in Table 2. There were no marked discrepancies in demographic data, anthropometric parameters, or any neurodevelopmental scores (all *p* > 0.05).

### 3.2. Primary Outcomes

As presented in Table 3, the primary outcome, pre-specified as FSIQ, did not show a statistically significant between-group difference after the 9-month intervention (adjusted mean difference: 1.05 points; 95% CI: −1.42 to 3.52; *p* = 0.400).

In the analysis of cognitive subdomains, a significant treatment effect was observed for PSI. After adjustment for baseline scores and clustering by class, the intervention group achieved a PSI score that was 5.91 points higher (95% CI: 1.88 to 9.93; *p* = 0.004) than the control group at the end of the study (Table 3). No significant between-group differences were found for the Verbal Comprehension Index (VCI), Visual Spatial Index (VSI), Fluid Reasoning Index (FRI), or Working Memory Index (WMI) (all *p* > 0.05).

### 3.3. Secondary Outcomes

#### 3.3.1. Growth and Development Indicators

**Figure 3:**
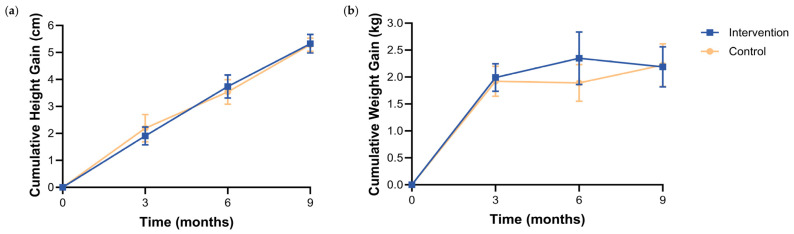
Growth outcomes over the 9-month intervention. (**a**) Cumulative height gain (cm). (**b**) Cumulative weight gain (kg). Measurements were taken at baseline (0) and at months 3, 6, and 9. Values are presented as means with 95% CIs. Between-group differences were tested at each time point using independent-samples *t*-tests; no significant differences were detected (all *p* > 0.05). Abbreviations: CIs, confidence intervals.

During the 9-month intervention period, cumulative height and weight gain increased progressively throughout the study in both groups (Figure 3). Inter-group variations in these gains were non-significant throughout the study period (all *p* > 0.05; Figure 3), suggesting that normal physical growth was sustained by both milk formulas.

#### 3.3.2. Blood Safety Parameters

Hematological and biochemical safety parameters are detailed in Table 4. At baseline, significant disparities were observed between the two arms regarding AST, CREA, and UA (all *p* < 0.05). By the end of the 9-month trial, no inter-group variation reached statistical significance for any blood parameter (all *p* > 0.05). All measured values remained within the normal clinical reference ranges for children throughout the study period.

### 3.4. Adverse Events

Across the whole study duration, no adverse events of clinical significance were documented among the participants.

### 3.5. Gut Microbiota Analysis

**Figure 4:**
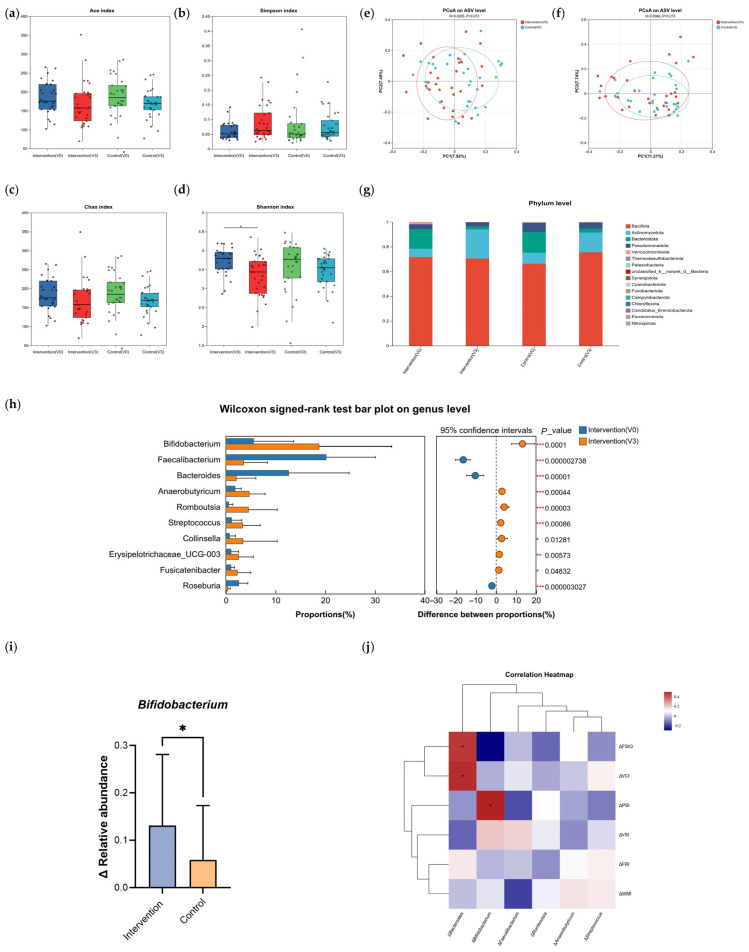
Effects of formula milk intervention on gut microbiota composition. (**a**–**d**): Alpha-diversity indices, including Ace (**a**), Simpson (**b**), Chao (**c**), and Shannon (**d**). (**e**,**f**): PCoA based on ASV profiles showing beta-diversity at baseline (**e**) and after 9 months of intervention (**f**). (**g**) Stacked bar plot of the relative abundances of major phyla. (**h**) Genus-level taxa showing significant pre–post changes within the Intervention group (Wilcoxon signed-rank test). (**i**) Between-group comparison of changes in relative abundance (Δ relative abundance) of *Bifidobacterium*. (**j**) Spearman correlation heatmap between changes in microbial relative abundances and cognitive outcomes. * *p* < 0.05; ** *p* < 0.01; *** *p* < 0.001. Abbreviations: ASV, amplicon sequence variant; PCoA, principal coordinates analysis; V0, baseline; V3, month 9.

To assess the influence of formula milk intervention on the gut microbiome, we performed sequencing analysis on 120 stool specimens (Figure 4). Alpha diversity analysis indicated a notable rise in the Shannon index in the intervention group post-intervention (Figure 4d, *p* < 0.05), while the Ace, Simpson, and Chao indices remained statistically stable (Figure 4a–c). Beta diversity, represented via PCoA utilizing Bray–Curtis distances, indicated no significant overall structural separation between groups after intervention (PERMANOVA, *p* > 0.05; Figure 4e,f). Consistently, no significant changes in the relative abundance of major phyla were observed in the intervention group post-intervention (Figure 4g).

At the genus level, paired analysis within the intervention group identified several taxa with significantly altered relative abundance after 9 months (Wilcoxon signed-rank test, *p* < 0.05; Figure 4h). The most notable change was a significant increase in the genus *Bifidobacterium*. This increase was further confirmed by between-group comparison of the change (Δ relative abundance), which was markedly higher in the intervention group relative to the control arm (Mann–Whitney U test, *p* < 0.05; Figure 4i).

To investigate possible links between microbial shifts and cognitive outcomes, Spearman rank correlations were calculated between changes in cognitive indices and changes in the relative abundance of genera that showed significant pre-post alterations and maintained high abundance. A significant positive relationship was observed between the enriched relative proportions of the genus *Bifidobacterium* and greater improvement in PSI scores (Spearman r = 0.406, *p* = 0.026; Figure 4j). This specific association suggests a potential link between the intervention-induced modulation of this key microbial genus and the observed enhancement in cognitive processing speed.

### 3.6. Fecal Metabolite Analysis

**Figure 5:**
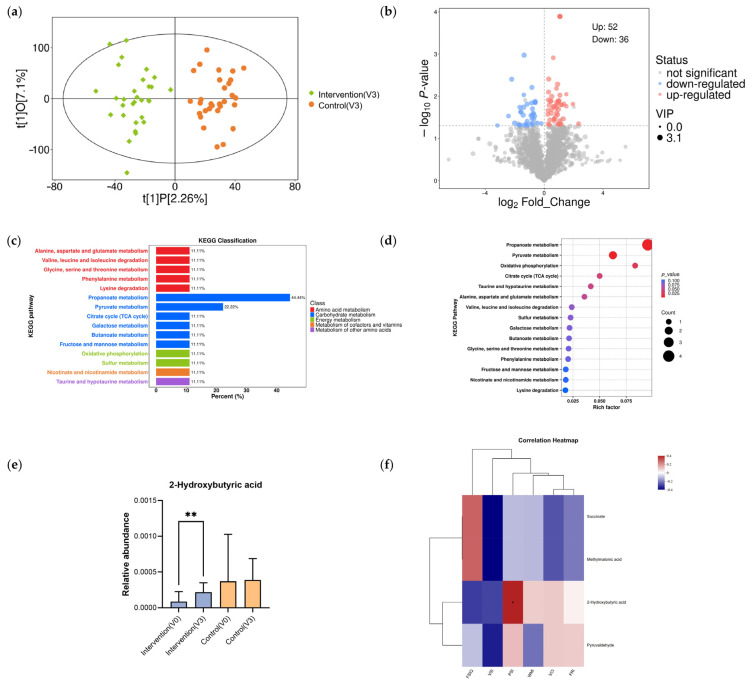
Effects of formula milk intervention on fecal metabolomic profiles. (**a**) OPLS-DA score plot for the Intervention and Control groups at month 9. (**b**) Volcano plot of differential metabolites between groups. (**c**) KEGG pathway classification for the differential metabolites. (**d**) Enrichment bubble plot for the differential metabolites. (**e**) Changes in the relative abundance of 2-hydroxybutyric acid before and after the intervention (** *p* < 0.01). (**f**) Spearman correlation heatmap between key differential metabolites and clinical cognitive measures (* *p* < 0.05). Abbreviations: KEGG, Kyoto Encyclopedia of Genes and Genomes; OPLS-DA, orthogonal partial least squares discriminant analysis.

Untargeted LC-MS metabolomic profiling was performed to assess the influence of formula milk intervention on the stool metabolome. Post-intervention, the OPLS-DA score plot revealed a clear divergence in metabolic signatures between the two groups (Figure 5a). Altogether, 46,789 metabolic features were captured. Utilizing thresholds of VIP > 1 and *p* < 0.05, we identified 88 metabolites that differed significantly between groups. As indicated by the volcano plot, 52 metabolites exhibited significant enrichment while 36 showed significant depletion in the intervention group relative to the control (Figure 5b). Further KEGG enrichment analysis highlighted that propanoate and pyruvate metabolism were the two most prominently impacted pathways following the intervention (Figure 5c,d).

Within these key pathways, we identified four differential metabolites: 2-hydroxybutyric acid (2-HB), pyruvaldehyde, succinate, and methylmalonic acid. Specifically, the relative abundance of 2-HB significantly increased in the intervention group after 9 months (*p* = 0.002), whereas no remarkable alterations were noted in the control arm (*p* = 0.901; Figure 5e).

To additionally examine the association between metabolic shifts and cognitive performance, Spearman correlation analysis was conducted between endpoint cognitive scores and the relative abundance of four differential metabolites. A notable positive association was observed between the post-trial PSI score and the relative levels of stool 2-HB within the intervention group (Spearman r = 0.366, *p* = 0.046; Figure 5f). This result suggests that the intervention-associated elevation of this microbial-related metabolite may be associated with the improvement in processing speed.

## 4. Discussion

This cluster-randomized trial demonstrates that a 9-month intervention with a multi-nutrient fortified formula, containing probiotics and prebiotics, significantly improved PSI in healthy preschool children, while no significant effect was observed on FSIQ. This improvement in cognitive performance coincided with a substantial rise in *Bifidobacterium* species and the stool level of 2-HB, which serves as a marker for propanoate metabolism. Both *Bifidobacterium* and 2-HB changes positively correlated with the degree of PSI improvement. To our knowledge, this provides the first integrated evidence from a RCT linking a composite nutritional intervention to improved specific cognitive function, beneficial gut microbial shifts, and functional metabolic changes in early childhood.

The differential response of FSIQ and PSI warrants careful interpretation. As a composite measure integrating multiple cognitive domains (e.g., verbal comprehension, fluid reasoning, working memory), FSIQ is shaped by an intricate interaction of genetic and sustained environmental factors [30]. Its stability might make it less susceptible to change within a relatively short intervention period, especially in a generally well-nourished population, a finding consistent with some prior trials [31,32]. In contrast, processing speed, measured by the PSI, reflects the efficiency of foundational neurocognitive operations. The preschool period (ages 3–6 years) is characterized by rapid development of neural infrastructure, particularly the myelination of white matter tracts, which is crucial for fast and synchronized information transmission [33,34]. Critically, the integrity of this myelination is closely linked to behavioral measures of processing speed [4,35]. This ongoing, experience-dependent maturational process may render neural efficiency metrics like PSI more responsive to supportive environmental inputs, including nutrition, during this specific developmental window [36]. Therefore, our results suggest that the nutritional intervention may have preferentially supported the optimization of these underlying, rapidly developing neural processes, rather than inducing a broad-scale shift in integrated cognitive ability within the trial timeframe.

The neural substrates that support processing speed, such as myelination, are highly plastic [37]. Their development and function are increasingly recognized to be regulated by a variety of systemic and microenvironmental factors. Beyond direct nutritional inputs, signaling molecules derived from the gut microbiota constitute one key category of factors that can remotely influence central nervous system development and function [18,38]. This theoretical framework helps contextualize our gut microbial findings. In this study, formula intervention markedly elevated the relative levels of *Bifidobacterium* within the intestinal tract, and this increase was positively correlated with improved PSI. Cohort studies indicate that higher *Bifidobacterium* abundance during infancy correlates with better subsequent neurocognitive functioning, supporting the potential critical role of this genus in neurodevelopmental processes [39,40,41]. However, these microbial findings should be interpreted cautiously. Although the relative abundance of *Bifidobacterium* increased significantly, no significant between-group differences were found in beta-diversity, and no significant changes in the relative abundance of major phyla were observed, suggesting that the overall microbiome shift was modest rather than global. Thus, the observed microbial changes are better regarded as genus-specific and exploratory. As a key microbial genus, *Bifidobacterium* is capable of synthesizing metabolic products, notably short-chain fatty acids, which are known to possess neuroactive properties and participate in gut–brain communication [42,43,44]. Therefore, the observed increase in *Bifidobacterium* abundance in this study may represent one potential pathway through which the nutritional regimen contributed to improved cognitive function in children, potentially mediated by microbial metabolites or other mechanisms.

The functional implications of this microbial shift were further elucidated by untargeted metabolomic profiling, which indicated that the intervention altered the fecal metabolite profile. KEGG pathway enrichment analysis revealed that differential metabolites were significantly enriched in propanoate metabolism. Notably, the level of 2-HB was markedly elevated and correlated positively with improvements in children’s PSI scores. 2-HB is understood to originate primarily from microbial degradation of amino acids in the gut [45]. Its increase likely indicates an enhanced microbial propionate synthesis pathway. Although 2-HB itself is not a typical gut–brain signaling molecule, propionate and other SCFAs produced via this pathway are well-established gut–brain communicators. They are involved in central nervous system modulation through neuro-immune signaling, which include modulate microglial function, maintain blood–brain barrier (BBB) integrity, and reduce neuroinflammation levels [46,47]. Therefore, the observed enrichment of *Bifidobacterium* and elevated 2-HB levels suggests that the nutritional intervention remodeled the gut environment in a way that may promote propionate metabolism. Whether these changes are directly linked or represent complementary aspects of a broader microbial shift, they collectively point to an upregulation of functional pathways capable of producing neuroactive metabolites, such as SCFAs. This upregulation represents a plausible mechanism by which the intervention could engage the gut–brain axis to support cognitive processing speed.

It should be clarified that the formula milk used in this study is a complex system containing multiple neuroessential nutrients (e.g., DHA, ARA) alongside probiotics and prebiotics. The direct role of DHA and ARA in neural structure development has been well-established [48]; vitamins and minerals are essential components for fundamental neural metabolism [49]. Therefore, the observed cognitive improvements in this study are likely attributable to the combined effects of two potential mechanisms. On one hand, DHA, ARA, and phospholipids directly provide essential structural support for the brain, aiding myelin formation and enhancing neural signal transmission efficiency. On the other hand, the unique combination of probiotics and prebiotics reshapes the gut microbiota structure centered on Bifidobacteria and the propionic acid metabolic network, thereby optimizing the metabolic environment of the microbiota–gut–brain axis and further promoting neurodevelopment. The findings of this study provide a more integrated perspective on how such composite nutritional formulations may support neurodevelopment.

When interpreting the current results, certain constraints of this research must be taken into account. First, the use of a composite formula precludes attribution of the observed effects to any single component (e.g., specific probiotics or DHA), which reflects the real-world application of such products but limits mechanistic specificity. Second, the cluster-randomized design, while pragmatic for implementation, may influence the precision of effect estimates despite statistical adjustment. Third, several important factors influencing cognitive development, such as socioeconomic status, parental education, diet, and screen time, were not fully controlled in the present study, which may have introduced residual confounding. Fourth, missing data were imputed using group mean values, which may have introduced bias and reduced the variability of the data. However, the overall missing rate was low (<5%), and the pattern of missingness was similar between groups. Fifth, the cognitive subdomain analyses (including PSI) were prespecified as secondary outcomes. Given their exploratory nature, no adjustment for multiple comparisons was applied. Therefore, these findings should be interpreted as hypothesis-generating and warrant confirmation in future studies. Sixth, the correlations observed, while informative, cannot establish causality between the gut microbiota/metabolite alterations and cognitive enhancement. Seventh, measurement of metabolites was limited to fecal samples; concurrent serum measurements would help clarify whether gut-derived signals enter systemic circulation. Finally, whether these cognitive and microbial gains are sustained over time remains unclear due to the absence of long-term monitoring. Future studies employing factorial designs, targeted biochemical profiling, and longer follow-up periods are needed to disentangle specific component effects, confirm the translational relevance of gut-derived signals, and assess sustainability.

Despite these limitations, our study has notable strengths. To our knowledge, it represents the first long-term RCT to employ an integrated multi-omics approach in assessing the effects of a fortified formula on both neurocognition and the gut ecosystem in healthy preschoolers. The key strength of this design is that it allowed us to move beyond reporting a standalone cognitive outcome. Instead, we observed a coherent pattern of change: a specific improvement in processing speed alongside concurrent increases in *Bifidobacterium* abundance and markers of propionate metabolism. Our findings provide valuable evidence supporting the conceptual “nutrient-gut-brain axis” in a pediatric population.

## 5. Conclusions

In this cluster-randomized trial, no significant between-group difference was observed for the primary endpoint, FSIQ. However, a 9-month intervention with a multi-nutrient fortified formula milk significantly improved PSI in preschool children. This cognitive benefit was paralleled by favorable changes in the gut ecosystem, notably an increase in *Bifidobacterium* abundance and elevated levels of 2-HB. Both of the gut changes correlated with the degree of cognitive improvement. The intervention demonstrated a favorable safety profile, with all children maintaining normal growth and biochemical parameters. By integrating multi-omics data, this study provides novel evidence that the benefits of a composite nutritional intervention may involve modulation of the microbiota–gut–brain axis, offering new insights for supporting neurodevelopment through early-life nutrition. However, because the PSI result reflects a domain-specific finding and no formal adjustment for multiple comparisons was applied to the cognitive subdomain analyses, this result should be considered exploratory and interpreted with caution. Of note, PSI was a secondary outcome; therefore, this finding should be considered exploratory and warrants further validation.

## Figures and Tables

**Table 1:** Nutritional and Functional Component Composition of Formula Milk Powder and Regular Milk Powder.

Nutrients	per 100 g	
	Formula Milk ^1,4^	Standard Milk ^1^
Energy	1924 KJ	1986 KJ
Protein	19.0 g	19.5 g
Fat	18.0 g	21.2 g
Carbohydrate	55.0 g	51.4 g
Sodium	400 mg	400 mg
Vitamin B2	0.30 mg	0.8 mg
Calcium	1210 mg	700 mg
Vitamin A ^2^	430 μg RE	- ^3^
Vitamin D ^2^	8.00 μg	-
Vitamin E	2.30 mg α-TE	-
Vitamin K ^2^	45.0 μg	-
Vitamin B1	0.75 mg	-
Vitamin C	30.0 mg	-
Niacin	2.70 mg	-
Folic Acid	140 μg DFE	-
Pantothenic Acid	3.2 mg	-
Choline	110.0 mg	-
Potassium	350.0 mg	-
Magnesium	30.0 mg	-
Iron	8.1 mg	-
Zinc	9.0 mg	-
Lutein	130 μg	-
Taurine	30.0 mg	-
Docosahexaenoic Acid	50.0 mg	-
Arachidonic Acid	60.0 mg	-
Lactoferrin	65.0 mg	-
CBP ^4^	≥150 mg	-
Yeast β-glucan	≥50.0 mg	-
*B. lactis* HN019	≥4 × 10^8^ CFU	-
*B. lactis* BL-99	≥2 × 10^8^ CFU	-
A2 β-casein	3.1 g	-

^1^ For both groups, each serving was prepared by mixing 25 g of milk powder with 160 mL of warm water and was administered twice daily (10:00 and 15:00) during the trial. ^2^ Vitamin A was added as retinyl acetate, vitamin D as vitamin D3 (cholecalciferol), and vitamin K as vitamin K2 (MK-7). ^3^ “-“ indicates that this item is not listed in the product’s nutrition facts panel. ^4^ Abbreviations: CBP, colostrum basic protein; CFU, colony-forming units; DFE, dietary folate equivalent; RE, retinol equivalent; α-TE, alpha-tocopherol equivalent.

**Table 2:** Baseline characteristics of participants.

Indicator ^1,2^		Intervention Group	Control Group	*p*
		*n* = 60	*n* = 60	
Sex, *n* (%)	Male	31 (51.7%)	33 (55.0%)	0.714
	Female	29 (48.3%)	27 (45.0%)	
Age (years)		5.48 ± 0.67	5.44 ± 0.48	0.752
Height (m)		1.12 ± 0.05	1.11 ± 0.06	0.122
Weight (kg)		18.98 ± 3.09	18.50 ± 3.31	0.408
BMI (kg/m^2^)		15.03 ± 1.61	15.05 ± 1.68	0.967
FSIQ		91.82 ± 8.37	90.60 ± 9.95	0.470
VCI		91.88 ± 11.33	91.37 ± 10.07	0.792
VSI		96.57 ± 9.69	93.22 ± 9.96	0.064
FRI		93.57 ± 9.25	91.70 ± 11.00	0.316
WMI		93.85 ± 9.80	93.38 ± 10.24	0.799
PSI		98.45 ± 10.99	96.28 ± 10.32	0.268

^1^ Values are reported as mean ± SD for continuous variables and *n* (%) for categorical variables. Analyses were performed in the intention-to-treat (ITT) set (*n* = 60 per group); missing observations were imputed using the within-group mean. *p*-values were obtained from an independent-samples *t*-test for continuous variables and a chi-square test for categorical variables. ^2^ Abbreviations: BMI, body mass index; FRI, Fluid Reasoning Index; FSIQ, Full Scale Intelligence Quotient; PSI, Processing Speed Index; VCI, Verbal Comprehension Index; VSI, Visual Spatial Index; WMI, Working Memory Index.

**Table 3:** Neurodevelopmental outcomes at baseline and after 9 months of intervention.

Indicator ^1,5^	Group	Baseline	9 Months	Adjusted Between-Group Difference ^2,3,5^ at 9 Months (95% CI)	*p* ^4^
FSIQ	Intervention Group (*n* = 60)	91.82 ± 8.37	93.83 ± 7.34	1.05 (−1.42, 3.52)	0.400
	Control Group (*n* = 60)	90.60 ± 9.95	91.68 ± 8.40	Reference	
VCI	Intervention Group (*n* = 60)	91.88 ± 11.33	92.94 ± 7.13	1.29 (−1.89, 4.48)	0.422
	Control Group (*n* = 60)	91.37 ± 10.07	91.66 ± 10.14	Reference	
VSI	Intervention Group (*n* = 60)	96.57 ± 9.69	95.90 ± 9.11	2.29 (−1.14, 5.72)	0.188
	Control Group (*n* = 60)	93.22 ± 9.96	93.89 ± 9.58	Reference	
FRI	Intervention Group (*n* = 60)	93.57 ± 9.25	95.48 ± 10.43	3.51 (−0.59, 7.61)	0.093
	Control Group (*n* = 60)	91.70 ± 11.00	91.75 ± 10.57	Reference	
WMI	Intervention Group (*n* = 60)	93.85 ± 9.80	95.50 ± 8.83	−0.83 (−4.44, 2.78)	0.651
	Control Group (*n* = 60)	93.38 ± 10.24	96.36 ± 10.96	Reference	
PSI	Intervention Group (*n* = 60)	98.45 ± 10.99	105.02 ± 11.81	5.91 (1.88, 9.93)	**0.004**
	Control Group (*n* = 60)	96.28 ± 10.32	99.07 ± 10.20	Reference	

^1^ Data are reported as mean ± SD. ^2^ The primary analysis for between-group comparison was performed using a linear mixed-effects model (ANCOVA approach), with the 9-month score as the dependent variable, adjusted for the corresponding baseline score and including a random intercept for class to account for cluster randomization. ^3^ The estimate represents the adjusted mean difference (Intervention minus Control) at 9 months with its 95% confidence interval. ^4^ Significant results (*p* < 0.05) are highlighted in bold. ^5^ Abbreviations: CI, confidence interval; FRI, Fluid Reasoning Index; FSIQ, Full Scale Intelligence Quotient; PSI, Processing Speed Index; VCI, Verbal Comprehension Index; VSI, Visual Spatial Index; WMI, Working Memory Index.

**Table 4:** Hematological and clinical biochemical safety parameters at baseline and after 9 months of intervention.

Indicator ^1,6^ (Unit)	Baseline (V0)	9 Months (V3) ^3^	*p* ^a,4,5^	*p* ^b,4^
Hematological Parameters				
HGB (g/L)				
Intervention Group ^2^	131.2 ± 7.8	135.0 ± 6.3 ***	0.699	0.497
Control Group ^2^	131.7 ± 7.4	135.9 ± 7.9 ***		
RBC (×10^12^/L)				
Intervention Group	4.8 ± 0.3	4.9 ± 0.3	0.851	0.584
Control Group	4.8 ± 0.3	4.9 ± 0.3 *		
MCV (fL)				
Intervention Group	82.3 (80.4, 84.0)	82.8 (81.5, 84.8) **	0.134	0.247
Control Group	83.3 (81.1, 85.6)	83.7 (82.4, 84.9)		
MCH (pg)				
Intervention Group	27.2 ± 1.2	27.6 ± 0.9 **	0.818	0.862
Control Group	27.3 ± 1.2	27.7 ± 1.1 **		
MCHC (g/L)				
Intervention Group	329.8 (326.3, 336.0)	330.9 (327.3, 335.0)	0.132	0.281
Control Group	329.0 (324.0, 333.0)	330.5 (328.0, 333.0)		
WBC (×10^9^/L)				
Intervention Group	6.3 (5.4, 7.3)	7.8 (6.5, 8.8) ***	0.407	0.335
Control Group	6.5 (5.6, 7.4)	7.7 (6.3, 8.6) ***		
Lymphocytes (%)				
Intervention Group	48.7 (42.1, 53.4)	41.2 (34.5, 47.3) ***	0.967	0.985
Control Group	47.7 (43.5, 54.3)	41.3 (34.7, 46.9) ***		
Neutrophils (%)				
Intervention Group	41.0 (36.5, 47.0)	48.7 (43.2, 54.8) ***	0.962	0.652
Control Group	41.7 (36.5, 46.0)	48.5 (41.0, 54.1) ***		
PLT (×10^9^/L)				
Intervention Group	326.0 ± 67.6	339.1 ± 51.9	0.119	0.854
Control Group	344.5 ± 61.5	341.0 ± 61.1		
Liver Function				
ALT (U/L)				
Intervention Group	11.0 (9.0, 14.0)	13.0 (10.0, 14.0) ***	0.474	0.414
Control Group	11.0 (10.0, 13.0)	13.0 (11.3, 15.1) **		
AST (U/L)				
Intervention Group	27.0 (25.0, 29.0)	26.9 (25.0, 29.8)	**0.038**	0.367
Control Group	29.0 (25.3, 30.0)	27.9 (25.0, 30.0)		
GGT (U/L)				
Intervention Group	10.0 (9.0, 11.0)	15.0 (14.0, 16.0) ***	0.900	0.114
Control Group	9.9 (9.0, 11.0)	14.8 (13.0, 15.8) ***		
TBA (μmol/L)				
Intervention Group	2.2 (1.7, 3.3)	4.6 (3.2, 5.8) ***	0.799	0.761
Control Group	2.4 (1.7, 3.8)	4.6 (3.5, 5.9) ***		
ALB (g/L)				
Intervention Group	44.0 (43.0, 45.0)	44.9 (44.0, 46.0) **	0.311	0.085
Control Group	44.0 (43.0, 46.0)	45.3 (44.0, 47.0) **		
PA (mg/L)				
Intervention Group	216.1 ± 23.3	208.7 ± 18.8 **	0.603	0.458
Control Group	214.0 ± 21.9	205.7 ± 25.1 *		
CHE (U/L)				
Intervention Group	8641.0 (7910.8, 9231.8)	9357.0 (8380.0, 9949.8) ***	0.629	0.711
Control Group	8508.5 (7700.0, 9297.8)	9464.2 (8463.8, 10,083.5) ***		
Renal Function				
CREA (μmol/L)				
Intervention Group	28.0 (26.0, 31.0)	41.0 (38.0, 43.0) ***	**<** **0.001**	0.544
Control Group	32.9 (30.0, 36.0)	42.0 (38.0, 45.0) ***		
UREA (mmol/L)				
Intervention Group	3.5 (2.9, 4.1)	3.8 (3.3, 4.2) **	0.137	0.128
Control Group	3.7 (3.1, 4.5)	4.1 (3.3, 4.6) *		
UA (μmol/L)				
Intervention Group	253.5 ± 46.8	227.6 ± 38.6 ***	**0.007**	0.414
Control Group	278.1 ± 50.7	233.6 ± 41.5 ***		

^1^ Data are expressed as mean ± SD or median (25th, 75th percentiles), as appropriate. ^2^ Analyses were performed in the intention-to-treat (ITT) population (*n* = 60 per group), and missing values were imputed using the group mean. ^3^ *, **, and *** indicate within-group changes from baseline (paired *t*-test or Wilcoxon signed-rank test; *p* < 0.05, *p* < 0.01, and *p* < 0.001, respectively). ^4^ *p* ^a^ and *p* ^b^ refer to between-group comparisons at baseline (V0) and month 9 (V3), respectively (independent-samples *t*-test or Mann–Whitney U-test, as appropriate). ^5^ Baseline *p* values < 0.05 are shown in bold. ^6^ Abbreviations: ALB, albumin; ALT, alanine aminotransferase; AST, aspartate aminotransferase; CHE, cholinesterase; CREA, creatinine; GGT, gamma-glutamyl transferase; HGB, hemoglobin; MCH, mean corpuscular hemoglobin; MCHC, mean corpuscular hemoglobin concentration; MCV, mean corpuscular volume; PA, prealbumin; PLT, platelet count; RBC, red blood cell count; TBA, total bile acid; UA, uric acid; WBC, white blood cell count.

## Data Availability

The data presented in this study are available on request from the corresponding author due to privacy and ethical restrictions.

## Supplementary Materials

The following supporting information can be downloaded at: https://www.mdpi.com/article/10.3390/nu18071167/s1, CONSORT 2025 checklist item. Ref. [50] was cited in Supplementary Materials.

## Abbreviations

The following abbreviations are used in this manuscript:

16S rRNA	16S ribosomal RNA
2-HB	2-hydroxybutyric acid
AE	Adverse event
ALB	Albumin
ALT	Alanine aminotransferase
ARA	Arachidonic acid
AST	Aspartate aminotransferase
ASV	Amplicon sequence variant
BMI	Body mass index
CBP	Colostrum basic protein
CFU	Colony-forming units
CHE	Cholinesterase
CI	Confidence interval
CREA	Creatinine
DFE	Dietary folate equivalent
DHA	Docosahexaenoic acid
FRI	Fluid Reasoning Index
FSIQ	Full Scale Intelligence Quotient
GGT	Gamma-glutamyl transferase
HGB	Hemoglobin
KEGG	Kyoto Encyclopedia of Genes and Genomes
LC-MS	Liquid chromatography–mass spectrometry
MCH	Mean corpuscular hemoglobin
MCHC	Mean corpuscular hemoglobin concentration
MCV	Mean corpuscular volume
OPLS-DA	Orthogonal partial least squares discriminant analysis
PA	Prealbumin
PCoA	Principal coordinates analysis
PLT	Platelet count
PSI	Processing Speed Index
RBC	Red blood cell count
RCT	Randomized controlled trial
RE	Retinol equivalent
TBA	Total bile acid
UA	Uric acid
VCI	Verbal Comprehension Index
VIP	Variable importance in projection
VSI	Visual Spatial Index
WBC	White blood cell count
WMI	Working Memory Index
WPPSI-IV	Wechsler Preschool and Primary Scale of Intelligence, Fourth Edition
α-TE	Alpha-tocopherol equivalent
